# New Ways to Disinfect and Fill Dental Hard Tissues

**DOI:** 10.3390/jcm9051524

**Published:** 2020-05-18

**Authors:** Matthias Zehnder

**Affiliations:** Division of Endodontology, Clinic of Conservative and Preventive Dentistry, Plattenstrasse 11, CH-8032 Zürich, Switzerland; matthias.zehnder@zzm.uzh.ch

The main biological goal in clinical dentistry remains to prevent micro-organisms from exerting pathogenic effects by establishing non-commensal biofilms or entering the wrong niches. If this has already happened, treatments aim to alter or remove these microbial communities, thereby arresting or healing the concurrent hard and soft tissue lesions. This Special Issue in the Journal of Clinical Medicine sought to gather studies, thoughts, and knowledge by experts in the field on new ways to disinfect and/or fill dental hard tissues. Contributions covered a wide array of topics, from preventive and restorative dentistry to root canal disinfection and filling.

A most interesting paper in the context of preventive dentistry, which was performed in an established oral biofilm model, showed that the composition of growing biofilms can be altered by enzymatic treatment [1]. This may lead to new approaches for biofilm dispersal with oral hygiene measures, and attempts to alter dental biofilms towards a commensal, rather than pathogenic, composition.

When it comes to restoring teeth affected by caries, so-called “bioactive” materials are already in fashion, yet may still be improved upon. The search is on for composite materials that not only restore teeth, but offer an element of prevention against future lesions by the controlled release of ions. A biologically active particulate material that may be more suitable than CaSi cements as a filler component in light-curing resin-based materials is bioactive glass, especially the classic Hench Bioglass 45S5 [2]. Two papers included in this Special Edition dealt with this topic. The first study showed that the size of the bioactive glass particles incorporated in experimental methacrylate-based restorative materials (composites) has a distinctive impact on bioactive properties of the material that has been functionalized in this manner. Downsizing bioactive glass particles to a nano size improved the alkalizing potential of experimental composites with no negative effects on their fundamental properties [3]. A second paper by that group introduced an experimental bioactive glass with lower Na_2_O content, yet with network connectivity similar to that of the conventional 45S5 material, additionally containing CaF_2_ [4]. This material showed a capability of forming fluorapatite, and could thus be incorporated in restorative and orthodontic materials to equip these with a known caries-preventive element.

Once a carious lesion reaches the pulp space, materials that exert beneficial effects on the pulp wound and induce healing/dentin bridge formation are of interest. The systematic review by Bossù et al. [5] confirmed the concept of replacing non-biocompatible materials, such as formocresol, with hydraulic calcium-silicate cements for primary tooth pulpotomy. However, mineral trioxide aggregate (MTA) as the pivotal hydraulic calcium silicate cement in its original form has a long setting time, and is not easy for the clinician to apply in a controlled manner. A further study thus investigated a resin-modified, MTA-based material (TheraCal LC) that can easily be set using light curing, yet may have untoward effects due to its monomer content in regard to its effects on human deciduous tooth-derived dental pulp cells [6]. The results of that study showed that TheraCal LC made the pulp cells express genes involved in two enriched Kyoto Enzyclopedia of Genes and Genomes (KEGG) pathways, which may be associated with osteoclastogenesis and osteoclastic differentiation, suggesting that it may not be an ideal material for direct contact with the dental pulp. In the context of vital pulp therapy, one aspect has not received the attention it deserves: the lavage of the potentially infected pulp wound. This was highlighted in a qualitative systematic review [7]. Future randomized trials should assess the effects of a sodium hypochlorite solution for this purpose.

When a dental infection has reached the root canal space, disinfection becomes the key issue. One publication in this compilation dealt with the concept of improving electrochemical disinfection of the pulp space by using a boron-doped electrode [8]. While this idea is compelling, the complexity of the root canal system makes disinfection hard to achieve, even when the most modern of tools are employed. Mechanical root canal instrumentation still appears to be the core step in this process, as was shown in a clinical study on 20 patients with asymptomatic apical periodontitis [9]. For the first time in endodontic research, the authors applied a combination of DNA and rRNA amplification to illustrate bacterial presence and activity simultaneously.

Once the root canal system is disinfected, the question then becomes how to fill it to provide a bacteria-tight seal. Sealers based on hydraulic CaSi cements have recently gained a large number of users and are heavily pushed by their manufacturers, yet clinical results have been elusive. A group of researchers from Guy’s Dental Hospital (King’s College) in London published the first clinical study on this topic in this Special Issue [10]. The results with this new type of sealer in conjunction with a simplified single cone-based application method appear promising after one year, even under the scrutiny of a cone beam-computed tomography outcome assessment, yet there is still a need for long-term follow-up observations. An even more biological approach to fill root canals, however, may be to attract pluripotent cells by a suitable scaffold and grow back a functional pulp. Two papers in this Special Issue deal with that matter. In the first, a concept is presented to use engineered nanometric chitosan particles due to their dual effect. They can act against biofilm, and at the same time, appear to have a stimulating effect on the host response to infection [11]. A second paper explored the possibility to embed pulp-derived exosomes in a fibrin matrix [12]. Results showed that the use of these exosomes may be a way to avoid regulatory issues with allogenic stem cells, which are the gold standard in experimental pulp tissue engineering. Moreover, there appeared to be a synergy between the fibrin gel and the exosomes on attracting stem cells.

Taken together, the current compilation of manuscripts puts forward some interesting new ideas that could lead the way into the future of conservative dentistry. However, it has to be kept in mind that new clinical approaches, as fancy as they may sound, will not always be better than the therapeutic concepts we already have.
